# Biochar for pollution mitigation and renewable energy applications toward sustainability development

**DOI:** 10.1039/d5ra09050d

**Published:** 2026-01-27

**Authors:** Hai Bang Truong, Van Dien Dang, Akhil Pradiprao Khedulkar, Joemer Adorna, Wan-Ju Yu, Thi Ai Ngoc Bui, Thamilselvan Annadurai, Muhammed Arshad, Minh-Ky Nguyen, Le Kim Hoang Pham, Nguyen Chi Toan, Giang Thi Thu Hong, Reetu Saini, Hoc Thang Nguyen, Cong Chien Truong, Thai Van Anh

**Affiliations:** a Optical Materials Research Group, Science and Technology Advanced Institute, Van Lang University Ho Chi Minh City Vietnam truonghaibang@vlu.edu.vn; b Faculty of Applied Technology, Van Lang School of Technology, Van Lang University Ho Chi Minh City Vietnam; c Faculty of Biology and Environment, Ho Chi Minh City University of Industry and Trade 140 Le Trong Tan Ho Chi Minh 700000 Vietnam; d Department of Biomedical Engineering and Environmental Sciences, National Tsing Hua University Hsinchu 30013 Taiwan; e Department of Chemistry, School of Sciences and Humanities, SR University Warangal Telangana 506371 India; f Department of Vocational Studies and Skill Development, School of Lifelong Learning, Central University of Haryana Mahendergarh 123029 India; g Faculty of Environment and Natural Resources, Nong Lam University Hamlet 33, Linh Xuan Ward Ho Chi Minh City Vietnam; h Faculty of Applied Science and Technology, Nguyen Tat Thanh University Ho Chi Minh City 755414 Vietnam; i Faculty of Pharmacy and Nursing, Tay Do University 68 Tran Chien Street Can Tho City 900000 Vietnam; j Faculty of Chemical Technology, Ho Chi Minh City University of Industry and Trade 140 Le Trong Tan Ho Chi Minh City 700000 Vietnam; k NTT Hi-tech Institute, Nguyen Tat Thanh University Ho Chi Minh City Vietnam; l Nguyen Tat Thanh University Center for Hi-Tech Development Saigon Hi-Tech Park Ho Chi Minh City Vietnam; m HUTECH Institute of Applied Sciences, HUTECH University Ho Chi Minh City Vietnam tv.anh@hutech.edu.vn

## Abstract

In the context of fossil fuels polluting the environment and depleting energy resources, the need to find sustainable solutions becomes urgent; in which, biochar stands out thanks to its potential applications in the fields of energy and environment. Biochar is produced from biomass and possesses advantageous structural properties, such as high surface area, porosity, and diverse functional groups, as well as ease of synthesis and compatibility with a wide range of low-cost, renewable feedstocks. This review outlines key biochar production methods-thermal, chemical, and biological-and evaluates recent advancements that enhance its structure and performance. These findings show that engineered biochar exhibits strong capabilities in pollutant adsorption, heavy-metal immobilization, and wastewater treatment, with surface chemistry playing a decisive role in removal efficiency. Biochar is being widely used in sustainable energy technologies, from electrode fabrication to renewable fuel production, due to its cost and environmental advantages. This review summarizes the potential of decentralized biochar production models for waste management and circular economy, identifies current research gaps, and discusses opportunities for future expansion. In doing so, the paper highlights the role of biochar as a promising solution to environmental and energy challenges.

## Abstract 2

In the context of fossil fuels polluting the environment and depleting energy resources, the need to find sustainable solutions becomes urgent; in which, biochar stands out thanks to its potential applications in the fields of energy and environment.

## Graphical abstract

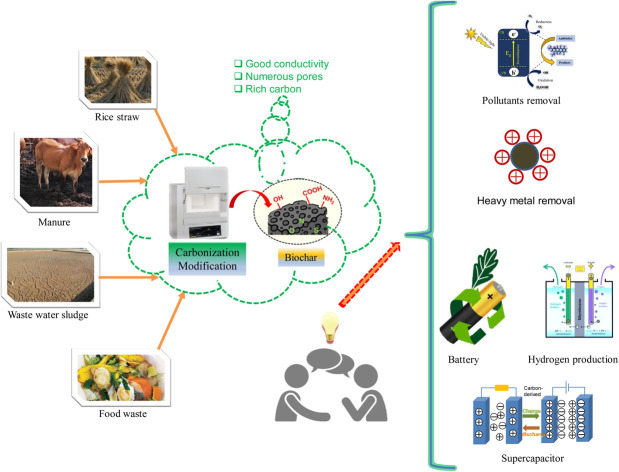

## 1. Introduction

Industrialization and modernization are causing the world to face resource depletion, greenhouse gas emissions and environmental pollution, while slowing this process has little long-term effect. In 2023, fossil fuels still account for 82% of global energy, continuing to promote climate warming. In response to such pressure, the need for sustainable solutions has spurred innovation in materials science, especially renewable materials and waste utilization.^1^ These materials are designed with the intention of simultaneously advancing the idea of sustainability and having acceptable performance qualities.^2^ Hence, the synthesis of renewable materials needs to be flexibly designed to suit many raw materials, many production techniques and many uses, while opening up diverse application pathways for the same type of material.^3^

A new class of materials called “waste-derived biochars” with a wide range of physicochemical characteristics has arisen as a promising avenue among the revolutionary advancements for various applications.^4^ Once considered waste, these materials are now being transformed into versatile resources for environmental restoration and energy production. As new ‘smart materials’, they contribute to shaping a sustainable future by turning waste into valuable products, while reducing ecological pressure and meeting energy needs.^5^ Waste-derived biochars are often synthesized from biomass, excreta, and crop residues through thermal decomposition or gasification processes in anoxic or inert gas conditions.^6,7^ Due to its high carbon content and unique surface structure, biochar becomes an attractive material for many applications, especially in the environmental and energy fields.^8^

The process of turning waste into resources begins with understanding the mechanisms of material creation and ends with mastering the factors that govern their synthesis and performance.^9,10^ The synthesis parameters determine the applicability of biochar, as factors such as temperature, feedstock and pyrolysis time strongly influence the carbon content, porosity and reactivity of the material.^11^ The effectiveness of biochar in various applications is determined by its structural properties, specifically its surface area, porosity, and conductivity.^12^ In addition, the thermo-chemical reactions during synthesis produce biochars with different degrees of porosity, doping, surface functionalization, and carbonization.^13,14^ Hence, an in-depth knowledge of these processes is necessary to maximize the performance of biochars for the particular requirements of energy and environmental applications.

**Fig. 1:**
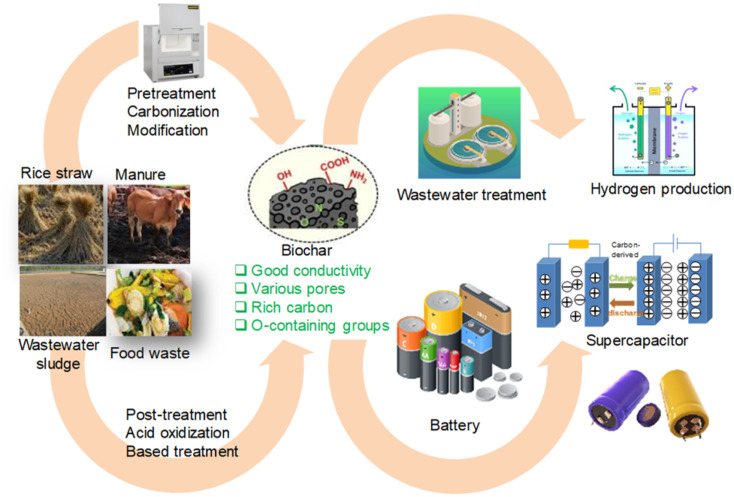
Biochar from multiple sources for environmental and energy applications.

Biochar from waste possesses many outstanding properties for environmental applications (Fig. 1), from treating polluted areas, adsorbing pollutants in water to adding nutrients to soil, contributing to improving agricultural productivity and sustainability.^15,16^

Biochar – a product of biomass pyrolysis – offers many benefits: soil improvement, long-term carbon storage and solid fuel. Pyrolysis also produces syngas and bio-oil, which can replace fossil fuels. Integrating biochar into the energy system contributes to promoting clean energy, decarbonization and a circular economy.^17,18^ One important mechanism that controls the performance of biochars is their sorption capacity, which is intimately related to their porosity, functional groups, and surface characteristics.^19^ In addition, the characteristics of biochar can be greatly influenced by the source of biomass, its chemical components, and the original cultivation location.^20^ Activation techniques, including physiochemical or biological activation/modification, determine the adsorption capacity and certain functional groups.^21^ Post-treatment procedures like impregnation or acid washing can further improve the qualities of biochar.^22,23^ Biochar moves through the water–soil–air cycle *via* leaching, runoff, erosion, and deposition,^24^ potentially causing health risks due to elevated PAH levels in amended soils. Wang *et al.* reported excessive benzo[*a*]pyrene concentrations in Chinese cabbage and pak choi.^25^ Over-application can also increase soil pH, salinity, PM emissions, and negatively affect invertebrates and agrochemical efficiency.^26^ In aquatic systems, biochar-bound pollutants may leach into water, threatening ecosystems and human health.^27^ Airborne biochar particles (*e.g.*, PM_10_) can cause respiratory issues upon inhalation.^28^ The safe use of biochar requires certified feedstock, appropriate application methods, and continuous monitoring. Scaling up production requires efficient, energy – efficient, and environmentally friendly pyrolysis technology. With its vast potential in energy and environmental applications – from storage electrodes, soil remediation, carbon capture, to water treatment-biochar still faces challenges in feedstock variability, process control, environmental impacts, standardization, and cost. Therefore, understanding the key factors is essential to optimize production. This review sheds light on the processes and variables that determine the formation of biochar from waste, highlighting the journey from waste to valuable resource.

## 2. Synthesis methods of waste-derived biochars

**Fig. 2:**
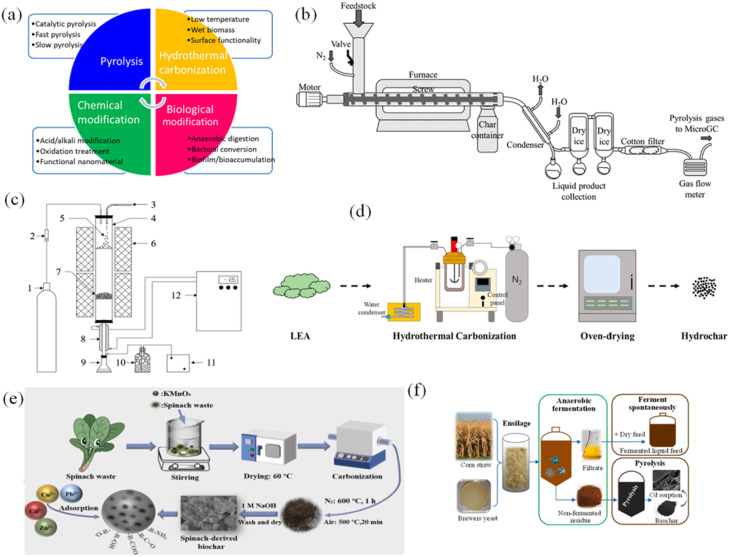
(a) The overview of synthesis methods of waste-derived biochars, (b) auger reactor system used for the slow pyrolysis experiments with wheat straw pellets,^39^ (c) biomass fast catalytic pyrolysis reaction system,^40^ (d) lab-scale setup for hydrothermal carbonization of lipid extracted algae,^43^ (e) activation of spinach waste biochar by KMnO_4_,^47^ (f) anaerobic fermentation of corn stalk and brewer yeast for biochar production.^48^ (Reproduced from ref. 39 Copyright 2022, ref. 40 Copyright 2021, ref. 43 Copyright 2018, ref. 47 Copyright 2024 with permission from Elsevier).

Fig. 2a shows the biochar synthesis methods such as pyrolysis, hydrothermal carbonization, and chemical–biological modification. Modified biochar holds promise for water treatment, soil remediation, energy storage and catalysis, but faces challenges of cost, standardization and environmental risks, while offering opportunities from waste streams and climate policy.^29^

### 2.1 Pyrolysis

Pyrolysis is the decomposition of biomass in a limited oxygen environment (250–900 °C), breaking down cellulose, hemicellulose, and lignin through depolymerization, fragmentation, and cross-linking.^30^ This process produces solid biochar and releases volatile gases, while obtaining valuable products such as bio-oil, biogas and heat.^31^ During pyrolysis, volatile gases continue to undergo cracking, reforming, and polymerization, which determine gas composition, PAH formation, and biochar structure. Controlling these gas-phase reactions is key to optimizing the product and tailoring material properties to the application.

Pyrolysis proceeds through two main mechanisms: primary and secondary. The primary mechanism involves the thermal breakdown of chemical bonds in the feedstock and the release of volatiles due to heat exposure.^32,33^ The secondary mechanism involves further transformation of unstable intermediates through cracking (producing lower molecular weight compounds) or recombination (leading to more complex inert or volatile molecules).^32,34^ Pyrolysis processes are often described by kinetic models, from first-order models to DAEM (Distributed Activation Energy Model), which aim to predict the decomposition rate and product distribution based on temperature and time in primary pyrolysis.^35^ Secondary pyrolysis is described by sequential or parallel reaction models, which optimize furnace design and product control, where temperature strongly influences biochar properties. Table S1 provides a comprehensive overview of the characteristics, advantages, and limitations of various heating-based biochar production methods.

Several factors influence the products obtained from pyrolysis, including process temperature, residence time (RT), biomass type, and heating rate.^36^ Among these factors, temperature is a crucial operating condition that determines product efficiency.^37^ Pyrolysis at low temperature (<450 °C) and slow heating gives high char yield, while rapid heating >800 °C produces more gas, ash and loss of surface functional groups.^38^ Slow pyrolysis, with its longer RTs and slower heating rates, is more likely to result in higher biochar ratios (Fig. 2b).^39^ In contrast, fast pyrolysis, characterized by short RTs and high heating rates, tends to yield higher ratios of oils (Fig. 2c).^40^ As pyrolysis temperature increases, the skeletal density generally decreases because progressive devolatilization and aromatization promote the formation of micropores and internal voids. Conversely, the bulk density typically increases with temperature, as particle shrinkage, structural contraction, and increased carbonization result in tighter packing and higher mass per unit volume when measured in the aggregated state. High pyrolysis temperatures produce more porous biochar but reduce CEC and volatile content. As temperature increases, volatile compounds decompose rapidly, releasing gases and forming porous structures, reducing material density. Nonetheless, excessively high temperatures can cause the collapse of pores or the merging of carbon frameworks, potentially disrupting the balance between density and porosity in the resulting biochar.^41^ These factors collectively impact the unique characteristics of the resulting biochar, thereby determining its suitability for specific applications.

### 2.2 Hydrothermal carbonization

Hydrothermal carbonization (HTC) converts biomass into carbon-rich hydrochar at 180–300 °C through dehydration, polymerization, and carbonization reactions.^42^ In the HTC process, water not only acts as a reaction medium but also acts as a catalyst due to its high ion product and low dielectric constant under subcritical conditions. Water promotes hydrolysis, dehydration and decarboxylation, converting biomass into carbon-rich hydrochar. The properties of water determine the carbon content, functional groups, porosity and heating value of the hydrochar. HTC is distinguished by its low-temperature operation, processing wet biomass without drying and producing materials with improved surface finish (Fig. 2d).^43^ Ercan *et al.* have shown that HTC at 250 °C and 275 °C is an effective method for converting lignocellulosic biomass (LB) waste (plant-based material) into biochars and hydrochars.^20^ Similarly, Krysanova *et al.* have examined the production of biochars through HTC of sawdust and peat across various temperature value ranges.^44^ HTC allows mineral recovery and increased heating value of biochar, while processing wet biomass at low temperatures (120–200 °C) without drying, superior to other thermochemical methods.

HTC produces carbon-rich hydrochar for soil improvement and carbon storage but takes a long time to process and produces liquid by-products, while pyrolysis and gasification are faster but require dry biomass; hydrochar properties depend on temperature, residence time and composition.^45^ However, the extent of these changes also depends on RT – longer durations can enhance carbonization but may result in structural degradation or increased ash content.^45^ Biomass composition further modulates these effects: lignin – rich feedstocks tend to produce hydrochars with greater aromaticity and thermal stability, while carbohydrate – rich biomass yields more functionalized and reactive surfaces.^46^ These parameters interact in complex ways, and must be holistically optimized to tailor hydrochar properties for specific applications.

### 2.3 Chemical modification

Chemical activation can be performed on biomass before pyrolysis or on biochar after its formation. Depending on the application, the material is treated with acid, alkali, oxidizing agent or impregnated with functional groups. Alkali is often used to develop porosity, while acid helps to increase the adsorption capacity of positively charged pollutants.^49^ This mechanism is based on the creation of a strong bond between biochar and the pollutant through the distribution of opposite charges. Acid – base activation adds functional groups such as –COOH, –OH, –NH_2_, with pH-dependent protonation states. Below pH_pzc_, the positively charged surface adsorbs anions; above pH_pzc_, the negatively charged surface adsorbs cations.^50^ Ionic strength further modulates the thickness of the electric double layer: higher salt concentrations compress the diffuse layer, reducing long-range electrostatic repulsion and enabling closer approach of charged species to the biochar surface.^51^ Non-uniformly distributed functional groups—often concentrated at the edges of pores or defects—create highly charged adsorption ‘hotspots’ that determine the ability and selectivity to bind contaminants. Oxidation treatments, using agents such as O_3_, H_2_O_2_, (NH_4_)_2_S_2_O_8_, or KMnO_4_, introduce oxygen-containing groups, modify the carbon skeleton, and incorporate minerals. For example, KMnO_4_ acts as a strong oxidizing agent, reacting with carbon to produce reactive Mn oxides along with CO_2_, thereby promoting the development of porous structures and enhancing ion transfer and metal adsorption capabilities at high temperatures (Fig. 2e).^47^ Although effective, oxidation can weaken the carbon structure, reduce surface area and create chemical residues, increasing processing costs. Therefore, it is necessary to optimize the oxidation level to avoid material destruction and ensure effective functionalization.

Many chemicals can be used alone or in combination with acids/alkali to form biochar-based nanocomposites, which help to increase the functional groups and active sites. Agents such as ZnCl_2_ and K_2_CO_3_ are well dispersed in the biomass, promoting mesopore formation and enhancing the adsorption capacity.^52^ Metal oxide/hydroxide–biochar nanocomposites can be prepared by soaking biochar or biomass in metal salt solutions (FeCl_3_, MgCl_2_, Ni(NO_3_)_2_). This composite material combines the large surface area of biochar with the high – energy reactive sites of oxides/hydroxides, thereby enhancing adsorption through electrostatic interactions, ligand exchange, and surface complexation. After treatment, the material needs to be washed to remove residual chemicals, while considering the environmental impact of the modification method.

### 2.4 Biological modification

The biological modification has three main processes, including anaerobic digestion, bacterial conversion, and biofilm/bioaccumulation. The biological modification provides biochars with hydrophobicity, high cation-anion exchange and porous capacity, which are beneficial to the adsorption processes. Tao *et al.* synthesized biochar from corn stalk and brewer yeast by combining anaerobic fermentation and pyrolysis (Fig. 2f).^48^ The author expressed that ensiling fermentation greatly enhanced the surface area, and oxygen-containing functional groups, and kept mineral components in biochar. Moreover, the redox potential and pH of biochar changed after being digested anaerobically, which had higher efficiency than the pristine for reducing the heavy metal, cationic dyes, methyl blue dye, and phosphate in aqueous from anaerobic digestion of dairy waste residues, bagasse.^53^

Anaerobic digestion can increase the surface area, functional groups, and mineral content of biochar, but at the same time, it creates VOCs and secondary emissions that pose environmental risks. Minimizing risks requires optimizing operating conditions, gas capture-treatment, and feedstock pretreatment. Therefore, despite its functional benefits, the process needs to be tightly controlled to limit environmental impacts. Microorganisms form biofilms in and on the surface of biochar, improving adsorption and degradation of pollutants; for example, naphthenic acid degradation efficiency increased from 30% to 87%.^54^ A combination of *Alcaligenes faecalis* and *Casuarina equisetifolia* seed-derived biochar formed a biofilm that removed 87% of methylene blue at a dosage of 500 ppm in a packed bed bioreactor (PBBR).^55^

Biofilm modification of biochar increases adsorption and degradation, but excessive growth can clog pores and reduce performance. Moderate nutrient conditions, adequate moisture and aeration help control biofilm, while measures such as backwashing and regeneration maintain long-term effectiveness. The structure and function of microbial communities on biochar are influenced by pH, temperature, and nutrient retention and release. pH determines bacterial or fungal colonization; temperature regulates metabolic activity; while carbon, nitrogen, phosphorus, and trace element uptake create favorable or restrictive microenvironments for each microbial group.^56–58^ This demonstrates that biological modification of biochar has the potential to enhance its adsorption capacity and pollutant degradation efficiency.

Biochar synthesis methods vary significantly in their scalability, feasibility, and environmental-economic impact. Pyrolysis is the most mature technology, providing high throughput and valuable by-products, but requires feedstock drying and is energy intensive without integrated heat recovery. HTC operates at 120–200 °C, processes wet biomass, and is suitable for decentralized models, but has long reaction times and requires wastewater treatment. Chemical modification increases surface functionality but introduces chemicals and costs. Biological methods are environmentally friendly but have limited scale. Overall, pyrolysis and HTC present the most immediate paths to large-scale, sustainable deployment, while chemical and biological routes offer specialized advantages that require further techno-economic and life-cycle optimization.

## 3. Characteristics of biochar

Biochar properties are often evaluated using a variety of complementary techniques: BET measures surface area; SEM/TEM observes morphology; XRD identifies mineral phases; FT-IR and Raman analyze functional groups and carbon order; XPS provides surface elemental composition and chemical state information, limited to the top ∼10 nm; TGA assesses thermal stability; solid-state NMR provides in-depth structural information. Understanding the limitations of each method helps to accurately interpret application performance.

### 3.1 Chemical composition

**Fig. 3:**
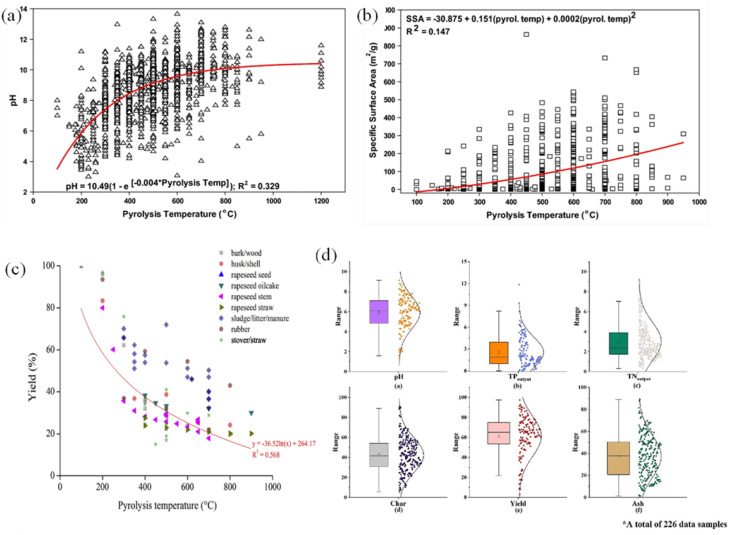
Effect of pyrolysis temperature on (a) pH (100–1200 °C), (b) specific surface area (100–1000 °C),^60^ (c) biochar yield;^61^ (d) distribution of data set of physical and chemical properties of 226 kinds of biochar.^59^ (Reproduced from ref. 59 Copyright 2023, ref. 61 Copyright 2018, with permission from Elsevier, ref. 60 Copyright 2020, with permission from Springer Nature).

The chemical properties of biochar were represented by elements (amount of C, H, N, S, and O), pH, electrical conductivity, cation exchange capacity, and functional groups. The kind of feedstocks and thermochemical process parameters decided the physical and chemical properties of biochar, which are illustrated in Table S2. Increasing pyrolysis temperature not only enhances pH value due to the loss of acidic functional group and the oxide, hydroxide minerals generation of cations (Fig. 3a) but also boosts SSA (specific surface area) because of the removal of hydro, oxygen, oils, and tar (Fig. 3b). With increasing pyrolysis temperature, biochar exhibits reduced yield (Fig. 3c), hydrogen and oxygen contents, and along with enhanced carbon enrichment. The H/C and O/C ratios thereafter dropped, suggesting that the biochar had improved carbonization and aromaticity and had less surface polarity. The biomass atomic ratios of O/C, H/C, (O + N)/C, and (O + N + S)/C decompose and escape as condensable and non-condensable gaseous components such as CH_4_, CO, H_2_, CO_2_, and other gases. Chen *et al.* statistically calculated the distribution of pH value, total nitrogen (TN), and total phosphorus (TP) in 226 biochar kinds (Fig. 3d).^59^ This result shows that the mean value of pH, TN, and TP followed by 6.2, 5.2, and 3.8, corresponding to 1.3, 0.83, and 0.5 of the standard deviations.

Biochar contains many elements (C, H, O, N, S, P…) and corresponding functional groups. The H/C and O/C ratios reflect the degree of carbonization: low H/C indicates a stable aromatic structure; low O/C indicates few oxygen groups, reduced polarity and increased hydrophobicity. These ratios help evaluate the structure, durability and adsorption capacity of biochar. The contents of oxygen and nitrogen, including functional groups, were determined in N/C, H/C, and O/C ratios. The ratios of O/C and H/C expressed the carbonization process of pyrolysis such as O/C ratios < 0.2, 0.2–0.6, and > 0.6 showed stable, slightly stable, and unstable biochars; H/C ratios < 0.7 have higher aromatic ring formation ratios > 0.7.^62,63^ The ratios of O/C and H/C expressed the carbonization process of pyrolysis such as O/C ratios < 0.2, 0.2–0.6, and > 0.6 showed stable, slightly stable, and unstable biochars; H/C ratios < 0.7 have higher aromatic ring formation ratios > 0.7.^60^

The effects of biomass source and pyrolysis temperature on the physicochemical characteristics of biochar are presented in Table S3. The elemental composition, ash content, and lignocellulosic structure of biomass are strongly dependent on the cultivation method, growth conditions, and harvest time, which in turn influence the surface area, porosity, carbon content, and ash properties of biochar. These factors determine the performance of biochar in fuel production, energy storage, and electrochemical applications. Biochar has an electrical conductivity of 0.002–23.8 dS m^−1^, higher than activated carbon, and a strong cation exchange capacity due to its carboxyl, hydroxyl, and amino groups. Aromatic structures and redox active sites promote electron transfer and support methane production, although high pyrolysis can cause loss of functional oxygen groups. The oxygen functional groups on biochar – carboxyl, hydroxyl, carbonyl, and phenolic – play a key role in its cation exchange capacity and electrochemical properties. They create a negatively charged surface, which helps hold cations such as K^+^, Ca^2+^, Mg^2+^, and NH_4_^+^ and increases nutrient retention in soil. At the same time, the quinone and phenolic groups participate in oxidation-reduction reactions, promoting electron transfer, making biochar suitable for energy storage and pollution treatment.^64,65^

### 3.2 Surface properties

Density, surface area, particle size, and porosity are the main physical properties of biochar. Density generally increases with pyrolysis temperature due to loss of volatile matter and formation of a graphite structure. The study reported that biochar production at 700 °C (1149 kg m^−3^) had a higher density than production at 450 °C (986 kg m^−3^) and feedstock (1043 kg m^−3^).^66^ The specific surface area (SSA) of biochar varies from 100 to 1000 m^2^ g^−1^, which depend on the pyrolysis process and kinds of feedstock, and it is an essential parameter in the adsorption process due to its relation with the pore size; the enhancement SSA of biochar can be conducted by chemical methods (alkaline, acid) or physical methods (milling, thermal). For example, the SSA of wheat straw increased from 6.9 to 130 m^2^ g^−1^ after using the ball-milled method.^67^ Biochar particle size can be divided into three kinds: particle (<0.25 mm), powder (0.25–1 mm), and granules (>1 mm), therein, the majority of the biochar surface area and effective molecular adsorption are contributed by micropores (pore size ∼2 nm).^68^ Small particle size increases surface area and porosity due to the exposure of many small pores and increased surface exposure, while large particles typically have a more highly interconnected pore structure.

Small pores determine SSA by creating a large internal surface. Smaller particles increase SSA but grinding too finely can destroy the pores, so a balance is needed between expanding the surface and keeping the internal structure.^69,70^ Agglomeration of biochar particles can reduce SSA and porosity due to van der Waals, capillary, or electrostatic forces that obscure the micro-mesopores. Conversely, agglomeration can also create large pores between clusters, increasing porosity but not necessarily improving the available surface area for adsorption or electrochemical applications.^71,72^

## 4. Waste-derived biochars for energy applications

### 4.1 Supercapacitor

**Fig. 4:**
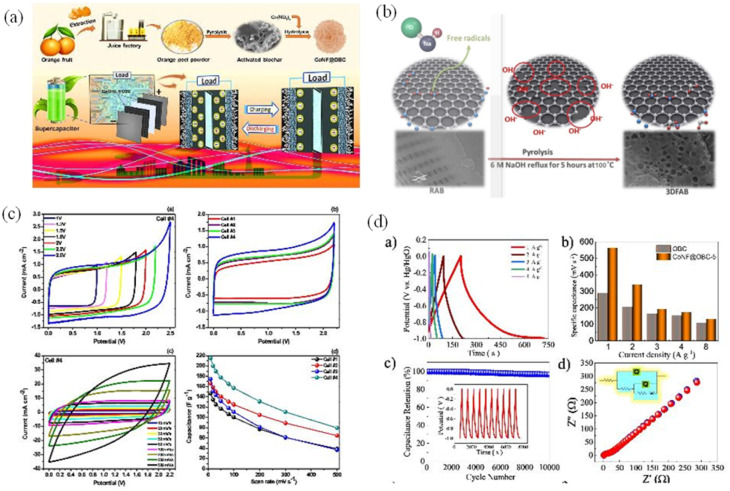
(a) Schematic illustration for preparation of Co(OH)_2_/orange peel biochar composite for application as an electrode material in supercapacitors,^74^ (b) possible mechanism of NaOH activation for the synthesis of 3D interconnected mesopores network of algal biochar,^75^ (c) electrochemical performance of the quasi-solid-state carbon supercapacitors/EDLCs [(a) CV of a typical cell (Cell#4) with gradually increasing voltage ranges recorded at a scan rate of 10 mV s^−1^, (b) comparative CVs of all EDLC cells at a scan rate of 10 mV s^−1^, (c) CVs recorded at different scan rates for Cell#4, and (d) variation of specific capacitance (*C*_sp_) as a function of scan rates for all the EDLC cells],^76^ (d) charge–discharge studies to analyze supercapacitive properties [(a) GCD plots for the as-prepared CoNF@OBC-4 electrode, (b) the capacitance of OBC and CoNF@OBC-4 as a result of various current densities, and (c) cycling stability of prepared CoNF@OBC-4, (d) calculated and measured impedance of CoNF@OBC-4 in the region of 100 kHz to 100 mHz are compared in the Nyquist plot of CoNF@OBC-4 (inset: the equivalent circuit)].^74^ (Reproduced from ref. 74 Copyright 2021, with permission from Nature, ref. 75 Copyright 2022, ref. 76 Copyright 2022, with permission from Elsevier).

Biochar from waste is becoming a potential solution, both improving the energy storage performance of supercapacitors and opening up a direction for sustainable waste management for a greener future (Fig. 4a). The hierarchical porous structure of biochar-based HPC creates a wide interface with the electrolyte, promoting charge storage. Meso- and macropores support rapid ion transport to the micropores, reducing mass transfer resistance, while micropores provide multiple active sites, increasing adsorption capacity and charge capacity.^73^

Biochar, a highly porous carbonaceous material, has attracted attention due to its outstanding electrochemical properties and potential to enhance supercapacitor performance. The large surface area and pore network enhance ionic interactions, thereby improving charge storage and capacitance (Fig. 4b). Certain highly polar oxygen functional groups, such as carboxyl and anhydride, can hinder ion transport, increasing resistance and reducing capacitance. In addition, unmodified biochars often have low porosity and low surface area, limiting their EDLC (electric double-layer capacitance) potential. Modern activation techniques have significantly improved porosity and surface area. Controlled pyrolysis converts waste into energy-rich carbon, but high ash content can hinder ion transport and reduce electrode performance. This approach also reduces pollution from traditional waste disposal. The porous structure of biochar creates an effective ion adsorption environment, which enhances the charge storage capacity in the supercapacitor. Chemical and physical activation processes further optimize the material properties, expanding the energy storage performance.^77^ This versatility allows researchers to tailor biochar-based supercapacitors to meet specific energy storage requirements, creating a range of applications that can benefit from its remarkable attributes (Fig. 4c). Integrating waste biochar into supercapacitors provides high power density, allowing for fast energy transfer – ideal for applications requiring burst power such as regenerative braking or renewable energy.^78^ Supercapacitors using waste biochar absorb and release energy quickly, making energy systems more flexible; while high cycle stability ensures long-term. This feature is essential for applications requiring long-term energy storage, such as grid-scale energy buffering (Fig. 4d). Durability and stable performance make supercapacitors an attractive solution for renewable energy.^79^

Biochar from waste not only promotes technological innovation but also supports sustainable development. By diverting organic waste from landfills and reducing emissions, this approach fits into the circular economy, offering a solution that combines environmental conservation and energy application.^80^ In the face of climate and sustainability challenges, this approach demonstrates the value of cross-sector collaboration. Sustaining collaboration requires a coordinated effort between academia, industry and government, with supportive policies and research networks that foster knowledge exchange and innovation.

### 4.2 Battery electrodes

Biochar is a promising material for batteries due to its high conductivity, porous structure, and large surface area. Ease of production, low cost, and renewable resources make it suitable for commercialization, while the ability to tailor functional groups and form composites with metal oxides enhances energy storage performance.

**Fig. 5:**
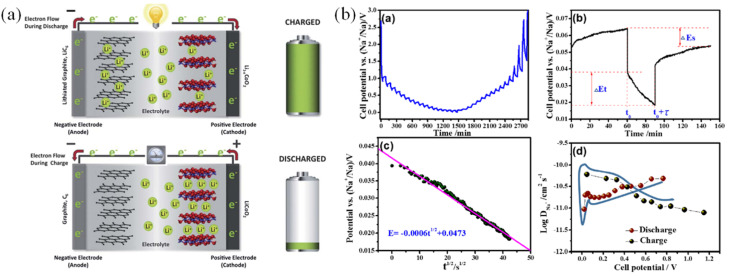
(a) A schematic illustration of the working principles of a Li_*x*_C_6_/Li_1−*x*_CoO_2_ lithium-ion cell,^81^ (b) performance of pine pollen biochar as electrode material for sodium–ion batteries [(a) galvanostatic intermittent titration technique (GITT) curve; (b) single GITT titration curve; (c) linear fitting of discharge voltages *E vs. τ*^1/2^ in a single GITT titration curve; (d) relationship between voltage and log DLi^+^ during the discharge and charge process].^82^ (Reproduced from ref. 81 Copyright 2012, with permission from Royal Society of Chemistry, ref. 82 Copyright 2018, with permission from American Chemical Society).

The Li-ions intercalate between graphitic planes in anode graphite with an interlayer spacing. The intercalation mechanism consists of 3 stages: intercalation (lithium ions are inserted into the layered frameworks of the graphite anode and layered oxide cathode, occurring without significant alteration to the structure. This “host–guest” process maintains the integrity of the electrodes, enabling excellent cycle stability), diffusion (Li^+^ ions move through the electrolyte and electrode materials by diffusion. The speed of this diffusion influences the battery's power output and charging rate), and electrochemical potential (the movement of Li^+^ ions is driven by the difference in chemical potential between the electrodes, which is directly linked to the battery's voltage). Energy density, cycle life, rate capability, safety, and stability have important implications for battery performance.^83,84^ Accordingly, Li-ions intercalate and de-intercalate on the graphite anode material during battery charging and discharging (Fig. 5a). Mining natural graphite is unsustainable, while biochar has emerged as an alternative material thanks to its porous structure, heteroatoms and defects that improve performance.^85^ The electrochemical performance of biochar electrodes strongly depends on structural defects and heterodoping (N, S, P, B). Defects such as edge sites or amorphous carbon domains create additional charge storage sites and improve ion diffusion. At the same time, heteroatoms modify electronic properties and increase conductivity, surface polarity and electrolyte wettability.^86^

Biochar offers advantages for lithium–sulfur batteries due to its large surface area and numerous functional groups, which help disperse sulfur and adsorb polysulfide, reduce the shuttle effect, and improve performance. However, the high surface area is prone to the formation of a large SEI (solid electrolyte interphase) layer in the early cycle. In addition, biochar also holds promise for sodium–ion, zinc–ion, and calcium–ion batteries (Fig. 5b). Emerging metal–air batteries, such as zinc–air and lithium–air batteries, have witnessed the utilization of various biochar materials as catalyst supports. Li *et al.* showed that nitrogen-doped biochar anodes achieved a high reversible capacity of 312 mAh g^−1^ after 200 cycles at a current density of 0.1 A g^−1^, exhibiting excellent rate performance by improving conductivity and a plentiful number of active sites.^87^ Biochar-derived carbon with hierarchical porosity maintained a capacity of 280 mAh g^−1^ after 100 cycles, which was credited to its efficient ion diffusion channels and robust structural stability.^88^ Given the involvement of oxygen reduction reaction (ORR) and oxygen evolution reaction (OER) in air batteries, the substantial variation in potential conditions poses a significant challenge.^89^ The stability of biochar helps the battery resist fluctuations and maintain performance.

### 4.3 Hydrogen production

Hydrogen is widely recognized as an alternative energy carrier, playing an important role in the neutralization of carbon and the minimization of greenhouse gases. Scientists are interested in hydrogen energy because it is less affected by outside factors than solar, wind, and tidal energy. Conventionally, hydrogen was mainly produced from natural gas and hydrocarbon (95%).^90^ Steam gasification of biomass can produce hydrogen but is expensive and emits CO_2_, so greener techniques are needed. Producing biohydrogen from renewable sources supports the goal of a ‘hydrogen economy’. Of the existing methods, water splitting is the most promising, with efficiency depending on the reduction of the overpotential in the HER (hydrogen evolution reaction) reaction.

**Fig. 6:**
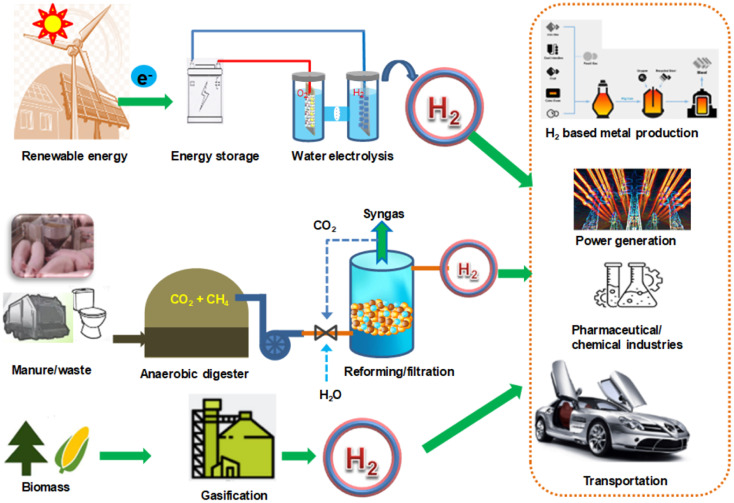
Hydrogen production sources and their applications.

Water splitting is a chemical process that decomposes water (H_2_O) into basic elements, including H_2_ and O_2_, requiring input energy. The reaction follows: 2H_2_O (l) = >2H_2_ (g) + O_2_ (g). The main water splitting methods includes electrolysis, photoelectrochemical, thermalchemical, biological. The hydrogen produced in this process is a clean energy source, storage material, and industrial feedstock that helps reduce carbon emissions. However, precious metal HER catalysts are expensive and unstable, prompting the search for alternatives. Biochar – based carbon is attracting attention because it is cheap, conducts electricity well, and utilizes waste, providing the dual benefits of energy production and pollution reduction. The development of the biochar field for hydrogen production has progressed at an impressive stage. Using biochar as a sacrificial electrode provides a sustainable and clean industrial-scale method for producing green energy carriers. Fig. 6 illustrates the hydrogen production sources, techniques, and applications. For example, Zhou *et al.* developed a pinwood-biochar sacrificial anode with high stability to actively assist water electrolysis for hydrogen production in concentrated alkaline electrolytes.^91^ Dewatered sewage sludge and food waste biochar have been used as a cathode for photocatalytic hydrogen generation from water.^92^ Despite its rich functional groups and high conductivity, biochar-based electrodes still exhibit lower HER overpotentials than many state-of-the-art materials. In HER, hydroxyl, carboxyl, and carbonyl groups provide proton adsorption sites; conductive carbon frameworks facilitate electron transfer; and when combined with metals/metal oxides, biochars provide a synergistic effect that stabilizes the catalyst. These mechanisms help reduce overpotentials by increasing the active area, improving adsorption, and promoting proton transfer.^93–95^

Heteroatom doping with transition metals is an effective way to reduce the overpotential and increase the HER performance of carbonaceous materials. For example, S–N-doped biochar from peanut roots achieved a starting voltage of only 27 mV *vs.* RHE (reversible hydrogen electrode), due to its large porosity and high electrochemical area. The authors proposed that the performance of the biochar is because of its rich porosity and high electrochemical area of 27.4 mF cm^−2^. Monteiro *et al.* used a carbon paste electrode (CPE) and a spongy material modified with cattle manure biochar to create an effective capacity for HER in acidic media.^96^ The electrode offered an overpotential of 0.34 V at 10 mA (*vs.* RHE) between the first and last analyses and high stability (200 h) during 1000 linear scanning cycles. These studies prove that biochar has the potential to be a plentiful alternative catalytic electrode for electrochemical reactions to achieve low-carbon hydrogen.

### 4.4 Bio-fuel production

**Fig. 7:**
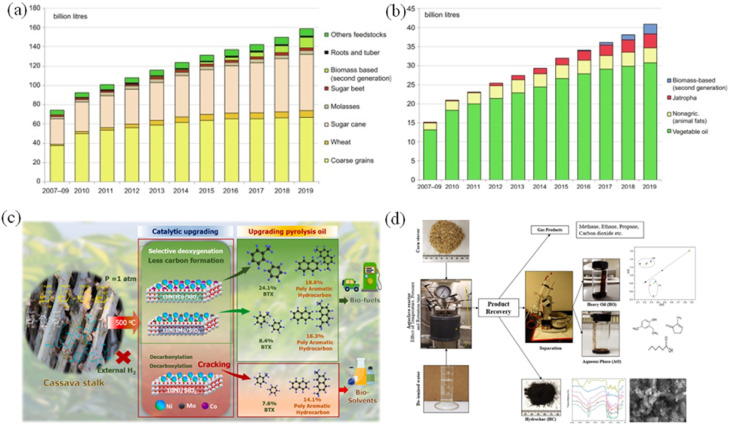
(a) Ethanol production yields (2007–2019), (b) biodiesel production yields *via* biofuel processes (2007–2019),^97^ (c) the overview of fast pyrolysis of cassava stalks-based materials for oil and syngas production,^18^ (d) hydrothermal liquefaction of lignocellulosic biomass corn stover for biofuel production.^101^ (Reproduced from ref. 97 Copyright 2020, ref. 18 Copyright 2024, ref. 101 Copyright 2020 with permission from Elsevier).

Biofuel is generated from agriculture, agricultural waste, or living plant intermediates. Ethanol and biodiesel are the main groups of biofuel. Fig. 7a and b show that biofuel production increased per year (2007–2019) worldwide; for instance, ethanol production increased from 77 to 160 billion liters, and biodiesel production increased from 15 to 41 billion liters.

Biofuels are produced by mechanical, thermochemical, chemical and biochemical methods. Of these, pyrolysis is commonly used because it quickly decomposes biomass into syngas, bio-oil and biochar under anoxic conditions (Fig. 7c).^97^ Based on the origination of feedstocks, biofuel is classified into four generations: 1st, 2nd, 3rd, and 4th generations. In the first generation of biofuel, peanut oil and vegetable oil were used for engine operation in 1900 and 1930, and the feedstocks were sugarcane, palm oil, soybean, *etc.* However, the 1st biofuel (conventional biofuel) could not meet the energy demand, and the negative appearance on the ecosystem and environment led to 2nd biofuel (cellulosic ethanol). The 2nd biofuel is generated from lignocellulosic and waste feedstocks with primary sources from energy crops (miscanthus, wheat straw, *e.g.*). Echaroj *et al.* used tungsten-zirconia as the catalyst in the palm fiber pyrolysis process for biofuel generation and 14.3% of gas, 7.1% of bio-char, and 40.5% of bio-fuel were produced,^98^ and 40 wt% of bio-oil product obtained after Jatropha wastes (leaves and stems) undergo pyrolysis process combined with metal/activated carbon catalyst.^99^ For 3rd biofuel (algae biofuels), algae are the primary feedstock. Its advantages are CO_2_ consumption, diverse living environment, rapid growth, and high nutritional–fuel diversity. Bhushan *et al.* used four kinds of algae (*S. obliquus*, *C. minutum*, *C. vulgaris*, and *Chlorella sorokiniana*) to evaluate its biofuel production ability in wastewater and 0.98 mL g^−1^ of CH_4_ yield obtained from *C. minutum*.^100^ Biomass with high yield and low cellulose–lignin compositions, metabolically engineered algae, and changing gene of feedstock (yeast, fungi, microalgae, cyanobacteria) are used in 4th biofuel (future technology) by integrating the production of biofuels with the capture and storage of CO_2_*via* the process of oxy-fuel combustion, or by using genetic engineering or nanotechnology; fourth generation biofuels attempt to provide more sustainable production choices.

Second- and fourth-generation biomass from waste, non-food crops and algae is more sustainable, supports a circular economy and reduces environmental risks. Biofuel production also creates jobs, promotes rural development and improves ecosystems through forest regeneration and waste utilization (Fig. 7d). Table 1 summarizes various applications of biochar for energy production and storage.

**Table 1:** Applications of biochar for energy production and storage

Feedstocks	Synthesis method	Applications	Efficiency	Capacitance retention/reusability	Ref.
Lacquer wood	One-step H_3_PO_4_ activation	Supercapacitor	354 F g^−1^ at 1 A g^−1^	95.3% after 10000 cycles	102
Torreya grandis inner-shell	Carbonization	Supercapacitor	354 F g^−1^ at 1 A g^−1^	97% after 5000 cycles	103
Waste potato peel	Hydrothermal carbonization and chemical activation	Supercapacitor	323 F g^−1^	94.3% after 10000 cycles	104
Sugarcane bagasse	Hydrothermal and ZnCl_2_ and CO_2_ gas activation	Supercapacitor	193 F g^−1^ at 1 A g^−1^	80% after 10000 cycles	76
Tea leaf	Pyrolysis and NaOH activation	Supercapacitor	945 F g^−1^ at 1 A g^−1^	95% after 10000 cycles	105
Sugarcane bagasse	Carbonization/Microwave activation	Energy storage	323.6 mAh g^−1^ at 0.05 A g^−1^		106
River driftwood	Hydrothermal treatment/carbonization	Energy storage	270–300 mAh g^−1^		107
Mango peel	Carbonization/N, S doping	Energy storage	400 mAh g^−1^ at 0.1 A g^−1^		108
Sawdust of poplar, catalpa, pine, and elm	Pyrolysis (700 °C for 2 h in N_2_ at 10 °C per minute)	Hydrogen production	109.848 mmoL g^−1^		109
Bamboo	Microwave pyrolysis	Hydrogen production	50.93 vol%		110
Corn stover	Pyrolysis (800 °C for 4 h in N_2_ at 10 °C per minute)	Hydrogen production	90 N mL g^−1^		111
Rice husk	KOH activation → Composites with K_2_O/Ni (RHC/K_2_O-20%/Ni-5%)	Bio-fuel	Biodiesel yield: 98.2%	Reusability: more than 70% after 5 cycles	112
Coconut shell	Calcination with *in situ* KOH activation;@ 600 °C, 1 h, N_2_	Bio-fuel	Yield: 93.0%	Reusability 86.1% after 5 cycles	113
Potato peel	Thermochemical conversion	Bio-fuel	Oil conversion 97.5%		114

Waste biochar has outstanding physical and chemical properties, making it suitable for energy applications such as supercapacitors, batteries, and biofuels. The large surface area and porous network provide numerous ion adsorption and transport sites. Functional groups (–OH, –COOH, C=O) support oxidation-reduction reactions and increase pseudo-capacitance, while the degree of graphitization improves conductivity. The wettability and surface charge determine the interaction with the electrolyte. Biochar also maintains structural stability and can be enhanced by combining with metals or metal oxides.^115–117^

## 5. Waste-derived biochars for environmental remediations

### 5.1 Adsorption of heavy metals and organic matters

**Fig. 8:**
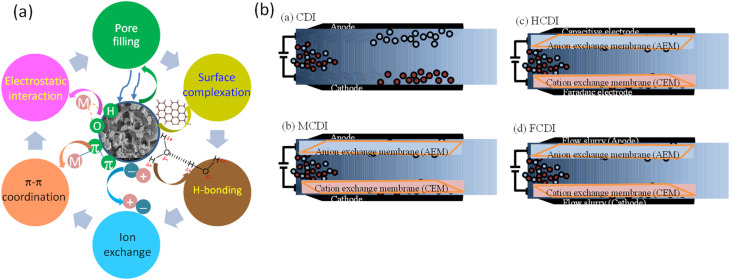
(a) Proposed mechanisms for contaminant removal by engineered biochar, (b) capacitive deionization (CDI) architecture diagrams of (a) conventional CDI, (b) membrane CDI, (c) hybrid CDI, and (d) flow-type CDI.

Various techniques have been proposed for removing heavy metals from water, including adsorption, chemically induced precipitation, ion exchange, electrochemically aided removal, and membrane separation, among others.^118–120^ Adsorption is an effective method, governed by Van der Waals forces, chemical bonds, π–π interactions, electrostatics, hydrophobicity and ion exchange (Fig. 8a). In addition to these mechanisms, heavy metal removal may also involve redox reactions (with biochar acting as a redox mediator to enhance the redox potential of metals), van der Waals forces, and precipitation (resulting from the presence of intrinsic metals in biochar and high pH conditions, facilitating metal precipitation within the biochar matrix). Table 2 shows the dominant characteristics of biochar in the organic matter and heavy metal removal process after the engineered biochar process.

**Table 2:** Biochar adsorption efficiency for the remediation of heavy metal and organic matter in aqueous environments^a^

Biochar	Properties	Pollutant	*C*_0_ (mg L^−1^)	Biochar dosage (g L^−1^)	pH	Equilibrium time (h)	Adsorption capacity (mg g^−1^)	Ref.
Zero-valent iron biochar	SSA: 113 m^2^ g^−1^, pore volume: 0.093 cm^3^ g^−1^	Cr(vi)	50	0.5	5.5	25	117.7	21
Chitosan-kiwi biochar	SSA: 3.3 m^2^ g^−1^	Cd(ii)	200	2.0	7.0	24	126.5	121
Magnetic biochar (Fe-BAB)	SSA: 66.5 m^2^ g^−1^, pore volume: 0.358 cm^3^ g^−1^	Cu(ii)	40	0.2	6.0	12	105.3	122
Oakwood	SSA: 332.9 m^2^ g^−1^, pore volume: 0.102 cm^3^ g^−1^	Cd(ii)	150	0.75	7.0	24	190.4	123
	—	Pb(ii)	150				392.2	
Bamboo	SSA: 220.1 m^2^ g^−1^, pore volume: 0.218 cm^3^ g^−1^	As(iii)	60	1.0	4.5	6	265.3	124
Boric acid-activated biochar	SSA: 119.6 m^2^ g^−1^, pore volume: 0.9 cm^3^ g^−1^	SMX	150	50	3.0	4	96.0	125
Magnetic Fe_2_O_3_/biochar	SSA: 431.6 m^2^ g^−1^, pore volume: 0.23 cm^3^ g^−1^	NFX	10.0	2.0	6	24	38.77	126
Biochar	SSA: 2457.3 m^2^ g^−1^, pore volume: 1.14 cm^3^ g^−1^	OTC	10	0.08	8.5	48	407.5	127
Seaweed biochar	SSA: 124.3 m^2^ g^−1^, pore volume: 2.1 cm^3^ g^−1^	CIP	10	0.5	6.5–8	12	93.65	128
Mg/N-spent coffee biochar	SSA: 115.6 m^2^ g^−1^, pore volume: 0.7 cm^3^ g^−1^	PO_4_^3-^	20	1	3–5	12	108.41	129

a *C*_0_: Initial pollutant concentration, SMX: sulfamethoxazole, NFX: norfloxacin, OTC: oxytetracycline, CIP: ciprofloxacin.

The key factors for the adsorption process include contact time, adsorbent dosage, adsorbate concentration, initial pH, and temperature. Organic pollutants and heavy metal removal are enhanced with the contact time increasing and reached to stabilize, which is assigned for saturation of adsorption sites. Also, the various absorbent–absorbates equilibrium determines different times. For example, Sulfamethoxazole (SMX) was rapidly adsorbed in the first 90 min and reached equilibrium at 120 min with orange peel biochar and coffee grounds, while the corresponding chitosan composites required 360–390 min to reach maximum capacity.^130^ The optimum biochar dosage determines the treatment efficiency, as increasing the material content provides additional adsorption sites, while high pollutant concentrations reduce the efficiency. Chu *et al.* stated that *o*-chlorophenol removal efficiency declined from 96.2 to 67.3%, corresponding to concentration enhancement from 10 to 150 mg L^−1^ with MgO-tea waste biochar due to the limit of surface area and active sites.^131^

pH is a significant factor in the adsorption process because it can affect both the surface charges of adsorbents and the ionization formation of adsorbates, facilitating the electrostatic attraction or repulsion between biochar and contaminants. For example, the excellent Cr(vi) removal efficiency was a pH of 2, then gradually decreased to a pH of 8.0, and slight changes appeared in pH of 8–10 by KOH-activated porous biochar.^132^ This phenomenon was attributed to Cr(vi) ionization formation and pH_pzc_. HCrO^4−^ and CrO_4_^2−^ (anionic) are dominant species when pH < 6, while CrO_4_^2−^ (anionic) is more abundant when pH > 6. Similarly, the surface charge of biochar is positive when pH > 2.2 and negative when pH < 2.2.

High temperature promotes movement and increases adsorption affinity. The thermodynamic equation (Δ*G* = Δ*H* – *T*Δ*S*, where G: Gibbs free energy, H: enthalpy, *T*: temperature, and *S*: entropy) was used to evaluate the temperature effect. At the range of 25–45 °C, levofloxacin adsorption was increased over NiFe_2_O_4_/biochar, and Δ*G* had a negative value which stated that this process was the irreversibility, heat-adsorbing and spontaneous.^133^

### 5.2 Capacitive deionization for saltwater desalination

Recently, research has delved into investigating efficient electrode materials and improving upon CDI (Capacitive Deionization) technology itself, which combats the main issue of CDI: reduced ion removal.^134^ Conventional CDI mechanisms, along with modified CDI architectures, are shown in Fig. 8b. In conventional CDI, current is applied between two electrodes separated by an insulating layer; however, deionization efficiency is degraded due to the phenomenon of homoelectrolyte repulsion, which increases the energy required when cations of the same charge are charged simultaneously. MCDI, by integrating specialized ion exchange membranes for each electrode, minimizes the penetration of homoelectrolyte ions, thereby enhancing charging efficiency and significantly improving deionization capability.^135^ The hybrid CDI (HCDI) has also been developed, which merges CDI with the asymmetric capacitance system found in battery electrodes. HCDI employs two distinct electrode materials: one based on carbon and the other on a pseudocapacitive/battery-type material. Coupling redox electrodes with porous carbon-based ones helps with enhancing CDI efficiency.^136,137^ However, despite the advances in CDI processes, current electrode materials are still limited in the treatment of high salinity waters, and both conventional CDI/MCDI are interrupted by a desorption step due to the low adsorption capacity of the immobilized electrode.^138^ Therefore, the FCDI architecture was initiated by Jeon *et al.*^135^ By incorporating a slurry-type electrode that recirculates throughout the CDI process, FCDI achieves unlimited ion capacity, enabling continuous desalination. With the technology in its infancy stages, current research efforts are committed to addressing the hurdles to fully realize the feasibility of FCDI.^135,139–142^ The ability of biomass-derived activated biochar, its modifications, and activated biochar composites for CDI applications is expressed by saltwater water desalination, heavy metal removal, and ion and organic matter removal.

Sustainable biochar play the role as a potential alternative to porous coal due to its electrochemical performance and environmental advantages. For example, Li *et al.* have prepared biochar from chitin using KOH as an activator at 800 °C, producing a high surface area of 833.8 m^2^ g^−1^ and possessing low charge transfer resistance.^143^ It exhibited a SAC of 11.52 mg g^−1^ with an initial concentration of 160 µS cm^−1^ NaCl under 2V applied potential. Interestingly, a high charge efficiency of 87.23% after several cycles was observed, which could be possible at lower concentrations as it eliminates the loss of charge efficiency due to parasitic reactions. However, the storage capacity needs definite improvement to work in real-life conditions, so to enhance the performance of biochar as a CDI electrode, various methods such as activation, pretreatment, compositing/doping, and modifications were conducted. The specific configurations and relevant parameters are presented in Table S4. Adorna *et al.* composited MnO_2_ with coconut shell-derived-activated biochar for CDI applications.^144^ Manganese dioxide (MnO_2_) is a transition metal oxide with high theoretical capacitance (>1300 F g^−1^), but it lacks applicability due to its subpar conductivity. By combining with highly conductive activated biochar, the composite garnered an SAC of 68.4 mg g^−1^ at 1.2 V under 1000 ppm NaCl. Hu *et al.* prepared CoCO_3_O_4_/N-CNTs with CNTs originating from glucose, which is easily accessible from biomass precursors.^145^ Hence, future studies must consider the use of the FCDI process to ensure continual utilization, maximizing its efficiency as a desalination technology.

### 5.3 Heavy metal removal

Biochar can also be an effective electrode material for the removal of heavy metals (arsenic, chromium, mercury, cadmium, copper, zinc, and lead).^118,146^ CDI is superior to other treatment technologies because it does not use chemicals and does not generate waste, in addition to a concentrated stream that is easily neutralized, thereby expanding the potential application in heavy metal removal. Table 3 presents a summary of some recent research results on the application of CDI for heavy metal removal.

**Table 3:** Heavy metal CDI performance of selected biochar-based electrodes^a^

Material	Pollutant	Conc.	Removal efficiency	Potential (V)	Remarks	Ref.
Algal biochar	Cu^2+^	50 ppm	75–120 mg g^−1^	0.8–1.5	SAC with other heavy metals: Cd(ii) > Zn(ii) > Cu(ii) > Ni(ii)	120
N,P-doped algal biochar	Cu^2+^	200 ppm	92.95 mg g^−1^	1.0	99% SAC retention after 5 cycles	119
N-doped silk cocoon biochar	Zn^2+^	40 ppm	31.3 mg g^−1^	1.0	71.7% SAC retention after 10 cycles	147
AC	Pb^2+^	0.5 mM combined	32%	1.2 V	Cd^2+^ was inhibited by presence of Pb^2+^ and Cr^3+^	148
—	Cr^3+^		43%	—		
—	Cd^2+^		52%	—		
AC	V^5+^	1500 ppm	106.89 mg g^−1^	—	Box-behnken design incorporated for electrode preparation	149
Rice husk biochar/MnO_2_	As^5+^	10 ppm	48.15 mg g^−1^	1.2 V	Active filter-CDI hybrid	118
Sewage sludge biochar	Pb^2+^	100 ppm	∼90 mg g^−1^	0.9 V	FCDI, 1.83-fold increase compared to AC upon desalting	150
AC	Cu^2+^	96 ppm 210.5 ppm NaCl	50%	1.2 V	FCDI, 94% SAC retention after 24 h continuous operation	151

a AC – activated carbon.

Most studies used activated carbon, while agricultural biochar showed higher CDI performance. Truong *et al.* conducted a study on biochar from *Sargassum hemiphyllum*, a common algae found on the coasts of Taiwan.^120^ The algal biochar exhibited good Cu(ii) removal at 75–120 mg g^−1^ with varying pH, the presence of competitive ions, and various applied voltages. The increase in the applied voltage will lead to a higher flow of electrons, resulting in stronger electrostatic attraction forces and improved efficiency in removing metal ions.^152^ The *Sargassum hemiphyllum* biochar, possessing a very high surface area of 1367.6 m^2^ g^−1^, exhibited a very high specific capacitance of 531 F g^−1^ at 1 A g^−1^. Additionally, the doped nitrogen/phosphorus atoms introduced opportunistic structural defects that resulted in electron density differences.^119^ Having this modification in the electron donor and its electron density enhanced its electrical conductivity and improved its chemical stability. These properties were essential to produce excellent adsorption performance of 56.16 mg g^−1^ with 50 ppm Cu at 1.0 V and increasing to 92.95 mg g^−1^ when the initial Cu^2+^ concentration is spiked to 200 ppm. Huang *et al.* imposed activated carbon cloth and checked for competitive heavy metal removal through CDI by combining Cd^2+^, Cr^3+^, and Pb^2+^.^148^ The average removal rates were found in the order of Cr^3+^ > Pb^2+^ > Cd^2+^. This order correlates with the hydration radius of these ions, with Cr^3+^ having the largest hydration radius and valence state compared to the other two ions. Hence, Cr^3+^ showed better removal efficiency than the other two ions. Cuong *et al.* prepared biochar from rice husk and composited with MnO_2_ and employed filtration to oxidize As(iii) to As(v) and initially removed As(iii, v), and combined with CDI to improve arsenic removal.^118^ Garnering a very high redox transformation efficiency of arsenic at 94%, it drastically improved arsenic capture on the filter, with the active BC filter having 72 times better removal than pure biochar. Pairing the system with a CDI unit further reduced the arsenic concentration to 1 ppb with a very low energy consumption of 0.0066 kWh m^−3^, marking real life applications in achieving WHO guideline values for potable water. CDI technology is highly compatible with heavy metal – polluted water sources and can be synergistically combined with other treatment methods to enhance removal efficiency. As heavy metal concentrations are relatively lower than that of the demand for desalination applications, CDI shows significant potential for effective heavy metal removal.

### 5.4 Ion and organic matter removal

Some studies on CDI have integrated the investigation of the effect of major ions, such as organics and inorganics, on electrode performance.^118,120,147,153^ In addition, there is growing concern about nutrient runoff from agricultural fertilizers and industrial – community waste sources, where phosphates and nitrates need to be removed and recovered to avoid eutrophication and algal blooms.^154^ Eutrophication causes ecological degradation and reduces landscape value; therefore, water nutrient control is necessary to protect aquatic life.

In recent years, researchers have been exploring the application of CDI technology for the removal of phosphate and nitrate in water, achieving some notable progress in this area. Zhang *et al.* have expounded that phosphorus, even at low concentrations of 10 ppm, could already trigger eutrophication in natural water streams.^155^ The α-MnO_2_/HPC and PANI/HPC electrodes were readily wetted and easily accessible for electrolyte solutions and present high specific capacitance. Electrosorption experiments found the SAC in the MnO_2_/HPC-PANI/HPC CDI cell to be 0.65 mmol g^−1^ for NaCl, 0.71 mmol g^−1^ for MgCl_2_, and 0.76 mmol g^−1^ for CaCl_2_. This results in a selectivity order of Ca^2+^ ≥ Mg^2+^ > Na^+^, confirming preference for divalent cations. The higher hydration energy of Mg^2+^ (−1830 kJ mol^−1^) compared to that of Ca^2+^ (−1505 kJ mol^−1^) may hinder the intercalation and deintercalation of Mg^2+^ ions.^156^ This selectivity is attributed to the stronger binding strength of divalent cations within the cavity of MnO_2_, and the active sites. These results would give a glimpse of the applicability of biochar for selectively removing hardness ions and other ions from polluted water to which researchers are investigating integrative and novel methods to accomplish this objective.

## 6. Conclusion and prospects

Biochar has proven to be a highly promising material for environmental remediation and energy applications, offering advantages such as high surface area, tunable surface chemistry, and compatibility with diverse biomass feedstocks. Its potential as a sustainable solution for waste management and resource recovery is supported by recent advances in synthesis techniques and functional enhancements. Key factors influencing its performance include feedstock type and doping strategies, while thermochemical co-conversion processes allow for the transformation of various waste sources into high-quality biochar. These developments demonstrate biochar's capacity for improving pollutant removal efficiency and electrochemical performance.

For industrial-scale deployment, it is essential to optimize biochar production by enhancing conversion efficiency, minimizing processing steps, and avoiding toxic reagents. Additionally, reducing residual impurities will further improve biochar's functionality in practical applications. A deeper understanding of surface chemistry and molecular interactions-achieved through a combination of theoretical and experimental studies-will be critical in guiding future innovations. Going forward, integrating biochar into circular economy models and evaluating its role in large-scale biowaste treatment systems will be important steps toward realizing its full potential in sustainable environmental and energy solutions.

## Conflicts of interest

There are no conflicts to declare.

## Supplementary Material

RA-016-D5RA09050D-s001

## Data Availability

No primary research results, software or code have been included and no new data were generated or analysed as part of this review. Supplementary information: the SI contains additional supporting data for this study, including tables summarizing: characteristics, advantages, and disadvantages of the pyrolysis process (Table S1), the feature characteristics of biochar synthesized in various sources (Table S2), physiochemical characterizations of various biochars (Table S3), and capacitive deionization performance of biochar-based materials under different CDI architectures (Table S4), along with the corresponding references. See DOI: https://doi.org/10.1039/d5ra09050d.
