# Supplementary material for: Biochar for pollution mitigation and renewable energy applications toward sustainability development
Source: RSC Adv. 2026 Jan 27;16(7):5834–51. doi: 10.1039/d5ra09050d (PMC12837511; doi:10.1039/d5ra09050d)
Supplement: RA-016-D5RA09050D-s001 [file RA-016-D5RA09050D-s001.pdf]

Supplementary Information (SI) for RSC Advances.
This journal is © The Royal Society of Chemistry 2026

SUPPLEMENTARY DATA

Biochar for Pollution Mitigation and Renewable Energy Applications toward Sustainability Development

Table S1. Characteristics, advantages, and disadvantages of the pyrolysis process

Pyrolysis	Characteristics	Advantages	Disadvantages	Ref.
Slow pyrolysis	• Temperature: 300−700℃<br>• Residence time: <2 s<br>• Yield of biochar: 35%	• High yield<br>• Stable and rigid structure<br>• High porosity<br>• Cost-efficient<br>• Biomass diversity	• Long residence<br>• Low energy product<br>• Time-consuming<br>• Feedstock preparation<br>• Emissions release	[1]
Fast pyrolysis	• Temperature: 500−1000 ℃<br>• Residence time: hour-day<br>• Yield of biochar: 12%	• Short residence<br>• High carbon content<br>• High porosity<br>• Rapid process<br>• Biomass diversity	• Energy requirement<br>• Low biochar yield<br>• Emission control requirement	[2, 3]
Catalytic pyrolysis	• The presence of selective catalysts<br>• Common catalysts: metal oxides, mesoporous solid catalysts, noble metal catalysts, molecular sieve catalysts, etc.	• Short duration<br>• Quick heating and pyrolysis of the material<br>• Higher energy saving<br>• High-quality pyrolysis product<br>• Improved the conversion rate	• Need catalyst support<br>• Cost of catalysts (e.g., precious metals)<br>• Technical complexity<br>• Feedstock sensitivity	[4, 5]

Hydrothermal carbonization	• Temperature: 180−300℃<br>• Residence time: 1−16 h<br>• Yield of biochar: 50−80%	• Effective method<br>• It can operate at low temperatures<br>• High surface functionality<br>• Potential energy generation	• Required pressurized, expensive (high-cost), and complex systems<br>• Feedstock limitations<br>• High energy input	[6-8]

Table S2. The feature characteristics of biochar synthesized in various sources [9]

Parameters	Feedstocks			
	Aquatic	Plant	Manure	Municipal solid
Features	Protein, lignocellulose, more inorganic compositions, and water	Lignocellulose, numerous quantities, broad distribution space	High nutrients (N, P, and K), more inorganic compositions.	Heavy metal appearance, moisture
Yield biochar	High	Low	High	High
Density	-	Small	Middle	High
Pore morphology	High porosity	Long structure	Honey-comb structure	Layers or sheets structure
Cation exchange capacity	Low	High	Low	Low
pH value	Rising with high temperature			
Specific surface area	Low	High	Low	Low
Porosity	400-700 ^{o}C ranges have high lignin – low ash compositions and outstanding porosity.			
	Rising with high temperature as temperature < 800 ^{o}C			

Table S3. Physiochemical characterizations of various biochars

Feedstocks	Temp (°C)	Physical			Chemical					Ref.
		Electrical conductivity (dS m^{-1})	SSA (m^2 g^{-1})	Pore size (nm)	pH	C/N	O/C	H/C	Ash (%)	
Digestate	400	4.5	4	10	10.6	23.5	0.23	0.68	28.7	[10]
Pepper stalk	600	0.16	71.3	3.2	10.8	32.9	0.09	0.32	10.6	[11]
Sewage sludge	300	-	57.7	-	6.5	8	0.19	0.12	-	[12]
Pine sawdust	650	-	130	-	9.6	260	-	0.05	-	[13]
Bamboo	200	-	9.3	14.8	5.18	133	0.67	0.11	0.95	[14]
Activated sludge	500	0.47	41.8	-	8.01	5.49	-	0.07	66.5	[15]
Rice husk	600	-	-	-	9.45	77.4	0.26	0.61	27.6	[16]
Fruitwood	900	-	-	-	8.63	208	0.07	0.47	5.49	[17]

Table S4. Capacitive deionization performance of biochar-based materials under different CDI architectures.

Architecture	Concentration	Material	SAC (mg/g)	CE (%)	Remarks	Ref.
Traditional	160 µS cm^{-1} NaCl	Chitin-derived biochar	11.4	87.2	26.5 mg g^{-1} at 500 µS cm^{-1}	[18]
	10 mM NaCl	Algae-derived biochar	8.7	75	Higher SAC and CE when used as cathode	[19]
	1000 ppm NaCl	Coconut shell-biochar/α-MnO_2	68.4	23.8	19.5% ion removal at 1000 ppm after 120 min	[20]
	824 ppm	$CoCO_3O_{4/}$ n-doped CNTs	60.1		85.6% SAC retention after 20 cycles	[21]
MCDI	1000 ppm	Activated carbon	-	90.3	Investigated effect of oil in brackish feedwater	[22]
	600 ppm NaCl	SCBFA-derived biochar	22	95	19 mg g^{-1} SAC retained after 70 cycles	[23]
	10 ppm	Activated carbon	-	99.2	10 MΩ cm UPW obtainable at 1.5V	[24]
Portable	1500 TDS	Activated carbon	75-80%$^{+}$	-	1.89 kWh m^{-3} at 7 L min^{-1}, prefers higher flow rates for better CE	

Notes: SAC – salt adsorption capacity, CE – charge efficiency, CNTs – carbon nanotubes, SCBFA – sugarcane bagasse fly ash UPW – ultra-pure water, TDS – total dissolved solids

References

[1] F. Rego, H. Xiang, Y. Yang, J.L. Ordovás, K. Chong, J. Wang, A. Bridgwater, Investigation of the role of feedstock properties and process conditions on the slow pyrolysis of biomass in a continuous auger reactor, J. Anal. Appl. Pyrol. 161 (2022) 105378.

[2] K.B. Cantrell, P.G. Hunt, M. Uchimiya, J.M. Novak, K.S. Ro, Impact of pyrolysis temperature and manure source on physicochemical characteristics of biochar, Bioresour. Technol. 107 (2012) 419-428.

[3] C. Chang, Z. Liu, P. Li, X. Wang, J. Song, S. Fang, S. Pang, Study on products characteristics from catalytic fast pyrolysis of biomass based on the effects of modified biochars, Energy 229 (2021) 120818.

[4] H. Guo, X. Qin, S. Cheng, B. Xing, D. Jiang, W. Meng, H. Xia, Production of high-quality pyrolysis product by microwave–assisted catalytic pyrolysis of wood waste and application of biochar, Arab. J. Chem. 16 (2023) 104961.

[5] X. Yang, B. Cao, D. Jiang, S. He, C. Yuan, H. Li, S.R. Naqvi, S. Wang, Catalytic pyrolysis of guaiacol on Enteromorpha-based biochar: A combination of experiments and density functional theory, Fuel Process. Technol. 239 (2023) 107527.

[6] A. Funke, F. Ziegler, Hydrothermal carbonization of biomass: a summary and discussion of chemical mechanisms for process engineering, Biofuels, Bioproducts and Biorefining 4 (2010) 160-177.

[7] B. Ercan, K. Alper, S. Ucar, S. Karagoz, Comparative studies of hydrochars and biochars produced from lignocellulosic biomass via hydrothermal carbonization, torrefaction and pyrolysis, J. Energy Inst. 109 (2023) 101298.

[8] M. Sevilla, A.B. Fuertes, The production of carbon materials by hydrothermal carbonization of cellulose, Carbon 47 (2009) 2281-2289.

[9] Y. Li, M. Kumar Awasthi, R. Sindhu, P. Binod, Z. Zhang, M.J. Taherzadeh, Biochar preparation and evaluation of its effect in composting mechanism: A review, Bioresource Technology 384 (2023) 129329.

[10] M. Stefaniuk, P. Oleszczuk, Characterization of biochars produced from residues from biogas production, J. Anal. Appl. Pyrol. 115 (2015) 157-165.

[11] J. Lee, X. Yang, S.-H. Cho, J.-K. Kim, S.S. Lee, D.C.W. Tsang, Y.S. Ok, E.E. Kwon, Pyrolysis process of agricultural waste using CO2 for waste management, energy recovery, and biochar fabrication, Appl. Energy 185 (2017) 214-222.

[12] S. Ren, M. Usman, D.C.W. Tsang, S. O-Thong, I. Angelidaki, X. Zhu, S. Zhang, G. Luo, Hydrochar-facilitated anaerobic digestion: Evidence for direct interspecies electron transfer mediated through surface oxygen-containing functional groups, Environ. Sci. Technol. 54 (2020) 5755-5766.

[13] N.M.S. Sunyoto, M. Zhu, Z. Zhang, D. Zhang, Effect of biochar addition on hydrogen and methane production in two-phase anaerobic digestion of aqueous carbohydrates food waste, Bioresour. Technol. 219 (2016) 29-36.

[14] U. Choe, A.M. Mustafa, H. Lin, J. Xu, K. Sheng, Effect of bamboo hydrochar on anaerobic digestion of fish processing waste for biogas production, Bioresour. Technol. 283 (2019) 340-349.

[15] C. Yin, Y. Shen, R. Yuan, N. Zhu, H. Yuan, Z. Lou, Sludge-based biochar-assisted thermophilic anaerobic digestion of waste-activated sludge in microbial electrolysis cell for methane production, Bioresour. Technol. 284 (2019) 315-324.

[16] J. Zhang, F. Lü, C. Luo, L. Shao, P. He, Humification characterization of biochar and its potential as a composting amendment, J. Environ. Sci. 26 (2014) 390-397.

[17] F. Lü, C. Luo, L. Shao, P. He, Biochar alleviates combined stress of ammonium and acids by firstly enriching Methanosaeta and then Methanosarcina, Water Res. 90 (2016) 34-43.

[18] P. Li, T. Feng, Z. Song, Y. Tan, W. Luo, Chitin derived biochar for efficient capacitive deionization performance, RSC Adv. 10 (2020) 30077-30086.
[19] B. Li, X. Liu, A. Wang, C. Tan, K. Sun, L. Deng, M. Fan, J. Cui, J. Xue, J. Jiang, D. Yao, Biochar with inherited negative surface charges derived from Enteromorpha prolifera as a promising cathode material for capacitive deionization technology, Desalination 539 (2022) 115955.
[20] J. Adorna, M. Borines, V.D. Dang, R.-A. Doong, Coconut shell derived activated biochar–manganese dioxide nanocomposites for high performance capacitive deionization, Desalination 492 (2020) 114602.
[21] X. Hu, X. Min, X. Li, M. Si, L. Liu, J. Zheng, W. Yang, F. Zhao, Co-Co3O4 encapsulated in nitrogen-doped carbon nanotubes for capacitive desalination: Effects of nano-confinement and cobalt speciation, J. Colloid Interface Sci. 616 (2022) 389-400.
[22] Y.-J. Kim, J. Hur, W. Bae, J.-H. Choi, Desalination of brackish water containing oil compound by capacitive deionization process, Desalination 253 (2010) 119-123.
[23] J.J. Lado, R.L. Zornitta, I. Vázquez Rodríguez, K. Malverdi Barcelos, L.A.M. Ruotolo, Sugarcane Biowaste-Derived Biochars as Capacitive Deionization Electrodes for Brackish Water Desalination and Water-Softening Applications, ACS Sustain. Chem. Eng. 7 (2019) 18992-19004.
[24] J.-H. Lee, J.-H. Choi, The production of ultrapure water by membrane capacitive deionization (MCDI) technology, J. Membr. Sci. 409-410 (2012) 251-256.
